# DYRK2 controls a key regulatory network in chronic myeloid leukemia stem cells

**DOI:** 10.1038/s12276-020-00515-5

**Published:** 2020-10-16

**Authors:** Chun Shik Park, H. Daniel Lacorazza

**Affiliations:** Department of Pathology and Immunology, Baylor College of Medicine, Texas Children’s Hospital, Houston, TX USA

**Keywords:** Chronic lymphocytic leukaemia, Cancer stem cells

## Abstract

Chronic myeloid leukemia is a hematological cancer driven by the oncoprotein BCR-ABL1, and lifelong treatment with tyrosine kinase inhibitors extends patient survival to nearly the life expectancy of the general population. Despite advances in the development of more potent tyrosine kinase inhibitors to induce a durable deep molecular response, more than half of patients relapse upon treatment discontinuation. This clinical finding supports the paradigm that leukemia stem cells feed the neoplasm, resist tyrosine kinase inhibition, and reactivate upon drug withdrawal depending on the fitness of the patient’s immune surveillance. This concept lends support to the idea that treatment-free remission is not achieved solely with tyrosine kinase inhibitors and that new molecular targets independent of BCR-ABL1 signaling are needed in order to develop adjuvant therapy to more efficiently eradicate the leukemia stem cell population responsible for chemoresistance and relapse. Future efforts must focus on the identification of new targets to support the discovery of potent and safe small molecules able to specifically eradicate the leukemic stem cell population. In this review, we briefly discuss molecular maintenance in leukemia stem cells in chronic myeloid leukemia and provide a more in-depth discussion of the dual-specificity kinase DYRK2, which has been identified as a novel actionable checkpoint in a critical leukemic network. DYRK2 controls the activation of p53 and proteasomal degradation of c-MYC, leading to impaired survival and self-renewal of leukemia stem cells; thus, pharmacological activation of DYRK2 as an adjuvant to standard therapy has the potential to induce treatment-free remission.

## Leukemia: Wiping out hidden reservoirs of stem cells

A master regulator protein may represent a key vulnerability for the eradication of stem cell populations that drive recurrence in chronic myeloid leukemia (CML). These leukemia stem cells (LSCs) remain robust and proliferative even after treatment has wiped out other reservoirs of cancer. Chun Shik Park and Daniel Lacorazza at Baylor College of Medicine, Texas Children’s Hospital, Houston, Texas, USA, have reviewed current knowledge about the molecular mechanisms that promote LSC survival, focusing on a protein called DYRK2. This protein’s normal physiological function is poorly understood, but it appears to restrain the uncontrolled proliferation of LSCs. Reduced expression of DYRK2 in these cells may play a major role in unleashing their tumorigenic potential. Agents that transiently reactivate DYRK2 could therefore prove a valuable adjunct to therapy, by promoting the deterioration and death of these cancer stem cells.

## Introduction

**Fig. 1:**
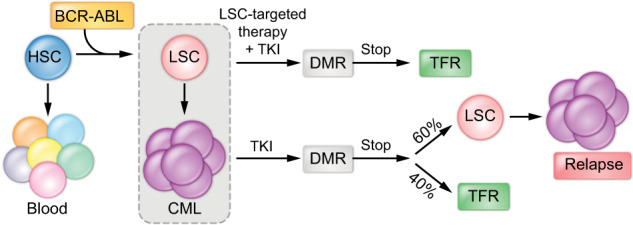
Pathobiology and treatment of chronic myeloid leukemia. The chromosomal translocation t(9;22) transforms a single hematopoietic stem cell (HSC) into a leukemia stem cell (LSC) through expression of the oncoprotein BCR-ABL1, which leads to the development of chronic myeloid leukemia (CML) via constitutive kinase activity. Treatment with tyrosine kinase inhibitors (TKIs) constitutes the standard of care and effectively induces a deep molecular response (DMR), although TKI discontinuation trials have shown that 60% of patients relapse, while 40% reach treatment-free remission (TFR). Future therapies to improve the induction of TFR should combine TKIs with LSC-specific drugs.

Chronic myeloid leukemia (CML) is a hematological malignancy that originates from hematopoietic stem cells (HSCs) by the expression of the constitutively activated BCR-ABL1 tyrosine kinase, which is a product of the chromosomal translocation t(9;22)^1–4^. This myeloid neoplasm is normally diagnosed in the initial chronic phase but can progress, if left untreated, to a lethal myeloid or lymphoid blast crisis through an accelerated phase. Although CML can be successfully managed by inhibiting BCR-ABL kinase activity with tyrosine kinase inhibitors (TKIs) (Fig. 1), most patients must remain on therapy indefinitely because this treatment cannot efficiently eradicate leukemic stem cells (LSCs)^5^. Imatinib was the first TKI that close to 20 years ago revolutionized the management of an incurable disease, and later, more potent second-generation (dasatinib, nilotinib, and bosutinib) and third-generation (ponatinib) TKIs were developed to overcome resistance to imatinib. Discontinuation trials in patients who have maintained complete molecular remission for at least 2 years have shown some success, with 40% achieving treatment-free remission after withdrawal of TKI therapy; however, 60% of patients unfortunately relapse (Fig. 1)^6–10^. This clinical evidence highlights the presence of a leukemic population with stem cell properties that is able to self-renew, resist pharmacological inhibition of BCR-ABL1 with TKIs, and initiate and sustain leukemia. This evidence also indicates that the development of LSC-directed therapy to prevent reactivation of BCR-ABL1-positive LSCs during the discontinuation of chemotherapy and to induce treatment-free remission (TFR) remains an unmet medical need. The development of LSC-specific drugs can potentially contribute to more effective TFR along with TKIs (Fig. 1). However, a bottleneck in the development of LSC-targeted therapy has been the lack of appropriate molecular targets that allow eradication of LSCs without altering normal blood cell production.

**Fig. 2:**
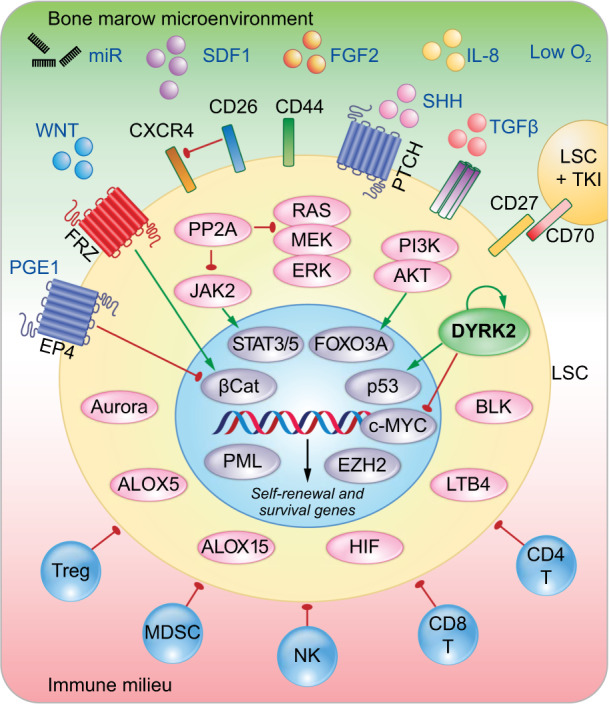
Genetic regulation of leukemia stem cells in CML. Diagram depicting the main regulatory pathways controlling LSCs. The survival and self-renewal of CML LSCs are controlled through a combination of cell-extrinsic and cell-intrinsic factors. Several intracellular events have been described in LSCs, such as activation of kinases, phosphatases, lipid metabolism, and oxygen sensing, which lead to nuclear regulation of genes involved in LSC self-renewal and survival via epigenetic and transcriptional regulation mechanisms. In addition, external factors, such as the bone marrow microenvironment (proleukemic signals) and immune surveillance (antileukemic cells), collectively interact to support or inhibit LSC survival.

CML LSCs are able to maintain self-renewal and survival through multiple mechanisms of resistance to TKI-mediated BCR-ABL1 inhibition, encompassing cell signaling (e.g., WNT, SHH, and TGFβ) with activation of kinases (e.g., PI3K/AKT, RAS/MEK/ERK, and JAK2/STAT3/5) leading to transcriptional activation through epigenetic and transcriptional regulation (Fig. 2). Figure 2 depicts a simplified view of the main mechanisms involved in LSC maintenance, which have been reviewed in excellent articles^11–13^. These mechanisms promote LSC survival via cytokines and growth factors within the bone marrow microenvironment. Conversely, accumulating evidence supports an important role of the immune environment during disease initiation and progression and, most importantly, in the maintenance of deep molecular remission (DMR) during TKI therapy. TKI-mediated inhibition of the oncoprotein BCR-ABL1 allows the restoration of immunosurveillance by effector T cells that keep at bay low numbers of LSCs and determines the success of TKI treatment discontinuation by preventing LSC reactivation and relapse. We recently reported that induced expression of the dual-specificity kinase DYRK2 either by genetic loss of the Krüppel-like factor 4 (KLF4) or inhibition of the ubiquitin ligase SIAH2, which is involved in DYRK2 proteolysis, abrogates the self-renewal and survival of CML LSCs^14^. The goal of this review is to highlight the main players in the regulation of CML LSCs and then to focus on DYRK2, which has been identified as a novel molecular target for the development of LSC-specific therapy.

## Regulation of self-renewal in CML LSCs

The mechanisms of CML LSC self-renewal and survival are regulated by diverse pathways that can depend on the signaling pathways activated by BCR-ABL1 (reviewed in refs. ^11–13^). Many factors in the bone marrow microenvironment that provide signals to CML LSCs for homing and survival (e.g., SDF1, FGF2, IL-8, WNT, miRNA, TGF-β, and STAT3/5) have been described, while immune cells, such as regulatory T cells, myeloid-derived suppressor cells (MDSCs), T cells, and—most importantly—natural killer (NK) cells, prevent disease progression and patrol residual LSCs in patients on TKI therapy (Fig. 2). Before discussing the identification of DYRK2 as a novel regulator of the self-renewal and survival of CML LSCs, we summarize known intrinsic pathways regulating the self-renewal and survival of CML LSCs (Fig. 2 and Table 1).

**Table 1:** Signaling pathways/molecules involved in the maintenance of CML LSCs.

Pathway/molecule	Role in LSCs	Reference
Wnt/β-catenin	Self-renewal, survival, and TKI resistance	^15,17,88,89^
Hedgehog	Self-renewal and survival	^18,19,90^
PI3K/AKT	Survival and TKI resistance	^20,21,23,91^
JAK/STAT5/3	Survival and TKI resistance	^24–28,30^
Ras/MEK/ERK	Survival and TKI resistance	^32–35^
Aurora kinases	Survival and TKI resistance	^92–95^
Blk	Self-renewal and survival	^66^
EZH2	Survival and TKI resistance	^49^
Fap-1	Survival and TKI resistance	^36^
HIFs	Proliferation and survival	^43,44^
PP2A	Self-renewal and survival	^38,39^
Alox5	Self-renewal and survival	^45^
Alox15	Self-renewal and survival	^46,47^
PML	Proliferation and TKI resistance	^48^
SIRT1	Survival and TKI resistance	^51–53^
BCL2	Survival and TKI resistance	^54–56^
PGE2	Self-renewal	^61^
KLF4/DYRK2	Self-renewal and survival	^14^

### Signaling pathways

The abrogation of LSC self-renewal and disease development caused by genetic loss of β-catenin in a BCR-ABL1-induced mouse model of CML and the synergistic effect of WNT inhibition and TKIs to control the leukemia burden by crippling LSC self-renewal support the role of the WNT/β-catenin axis in CML LSCs^15^. However, the WNT pathway may not be a suitable target for LSC eradication because β-catenin is also required to support self-renewal in normal HSCs^16^. In a different mechanism of WNT activation, TKI treatment induces CD70 expression on LSCs, which upon autocrine engagement with CD27 leads to nuclear translocation of β-catenin with activation of WNT target genes, further supporting TKI resistance^17^.

The canonical hedgehog (HH) pathway is activated through binding of a ligand (SHH, sonic hedgehog) to the PTCH receptor at the cell membrane, which relieves PTCH-mediated inhibition of smoothened (SMO) and initiates activation of a downstream pathway. This pathway mediates self-renewal in LSCs through SMO activation and the downstream transcription factor GLI1, which promotes MDM2-mediated proteolysis of p53 and activation of leukemogenic genes (e.g., MYC and cyclin D). Therefore, genetic or pharmacological inhibition of this pathway causes a loss of LSCs in mouse models without affecting HSCs and normal blood production. Drug combination studies using imatinib and an SMO antagonist (sonidegib) demonstrated inhibition of self-renewal capacity specifically in CD34^+^ cells from chronic-phase CML patients but not in normal CD34^+^ HSCs^18,19^.

Binding of growth factors to receptor tyrosine kinases (RTKs) leads to activation of the phosphoinositide-3-kinase B and AKT (PI3K/AKT) pathway. Activation of PI3K/AKT signaling by BCR-ABL1 is an important event in the maintenance of CML LSCs, as it leads to AKT-mediated phosphorylation and cytosolic retention of FOXO transcription factors and therefore decreased transcription of FOXO target genes involved in cell death^20,21^. Conversely, inhibition of BCR-ABL1 with TKIs promotes nuclear translocation of FOXO transcription factors, allowing transcription of FOXO target genes (e.g., the prosurvival factor BCL6); thus, inhibition of PI3K can sensitize LSCs to TKIs^22^. Consistent with crosstalk signaling, TGF-β can activate AKT, leading to FOXO3a cytosolic localization and supporting the survival of CML LSCs^23^. Consequently, the combination of TKIs with TGF-β inhibitors was effective in decreasing the progression of CML through eradication of LSCs.

The Janus kinase (JAK)-signal transduction and activator of transcription (STAT) pathway is involved in translating immune signals from cell membrane receptors (e.g., cytokine receptors) to the nucleus. Activation of the JAK/STAT pathway and activation of STAT5 by either JAK2 or BCR-ABL1 support the maintenance of CML LSCs^24,25^. As single agents, JAK2 inhibitors specifically target CML LSCs without affecting normal HSCs, whereas the combination of JAK2 inhibition with dasatinib treatment efficiently eradicated TKI-resistant CML LSCs; thus, pharmacological inhibition of JAK2 can synergize with TKI therapy^26,27^. Furthermore, a reduction in quiescent CML LSCs can be achieved without affecting normal stem cells by combining the JAK2 inhibitor ruxolitinib with nilotinib^28^. In addition to STAT5, STAT3 also plays a role in the survival of CML cells downstream of JAK2 signaling. Induction of STAT3 phosphorylation by imatinib was found to be enhanced during coculture with human bone marrow stromal cells, and inhibition of STAT3 in combination with imatinib targets TKI-resistant leukemic stem and progenitor cells^29,30^.

The RAS/MEK/ERK pathway is another kinase cascade activated by BCR-ABL1 in CML that translates signals from cell surface receptors to nuclear transcription factors. Inhibition of MEK5/ERK5 in CD26-positive cells reduced the culture repopulation ability, serial colony formation, and the formation of long-term culture-initiating cells (LTC-ICs), suggesting abrogation of self-renewal in leukemic stem cells^31^. The RAS/MEK/ERK pathway is also essential for resistance to TKI-induced apoptosis^32^. For example, MEK inhibition with PD184352 combined with treatment with the farnesyltransferase inhibitor BMS-2144662 increases apoptosis in TKI-resistant CD34^+^ CML cells^33^. In addition, targeting ERK1 and ERK2 by inhibiting the expression of GRB2, which is a potent activator of ERK1 and ERK2, has been shown to be effective in the treatment of CML patients with refractory or relapsed disease^34,35^.

Finally, tyrosine phosphatases involved in the regulation of kinase activation understandably play critical roles in CML LSCs given the diversity of kinase signaling pathways activated by BCR-ABL1. The tyrosine phosphatase FAS-associated phosphatase 1 (FAP-1) inhibits FAS-mediated apoptosis and stabilizes β-catenin through inhibition of GSK3β; therefore, inhibition of FAP-1 using a blocking tripeptide increased the response to TKIs and suppressed leukemia progression in mice transplanted with BCR-ABL1-mutant cells^36^. Protein phosphatase 2A (PP2A) is an important and ubiquitously expressed serine/threonine phosphatase that dephosphorylates cellular molecules such as AKT, p53, c-MYC, and β-catenin^37^. PP2A-activating drugs reduced the survival and self-renewal of quiescent CML LSCs—but not normal HSCs—through inhibition of JAK2 and β-catenin via a mechanism independent of BCR-ABL1^38^. On the other hand, pharmacological inhibition of PP2A with clinically tested inhibitors was shown to impair the survival of TKI-resistant stem/progenitor cells, sensitizing them to TKI therapy in BCR-ABL1-positive leukemia^39^. The clinical application of modulating PP2A activity needs to be further investigated.

### Cellular metabolism

In contrast to normal HSCs, which produce energy largely through glycolysis to support the quiescent state and self-renewing divisions, leukemic stem/progenitor cells rely instead on oxidative phosphorylation as a source of energy during homeostasis, as shown by a metabolomic study. Therefore, inhibition of mitochondrial translation with tigecycline induces cytotoxicity in CD34^+^ CML cells in combination with imatinib^40^. An interesting example of how LSCs evade chemotherapy through changes in metabolism driven by the microenvironment is the ability of TKI-resistant LSCs to lodge in a gonadal adipose niche where they undergo a metabolic switch to lipolysis and free fatty acid production^41^. A hypoxic microenvironment regulates the proliferation and survival of CML LSCs via hypoxia-inducible factor 1 (HIF-1) and reprograms cancer metabolism through activation of genes involved in glucose transport and glycolytic enzymes. LSCs cultured under hypoxic conditions were found to show increased HIF-1α-driven glycolysis as the main source of energy production^42^. HIF-1α is required for the maintenance of CML LSCs, and loss of HIF-1α was found to impair disease progression in a CML mouse model by inhibiting the cell cycle and inducing apoptosis in LSCs^43^. Inhibition of HIF-1 with acriflavine reduced the colony formation and long-term culture capacity of primary CML cells and decreased the number of LSCs in vivo without a significant effect on normal cells^44^. Because CML LSCs rely more on HIF-1 than do normal HSCs^43^, combination treatment with an HIF-1 inhibitor and a TKI impaired CML LSCs that were located in the hypoxic region in the bone marrow microenvironment while minimizing the effect on normal HSCs^43,44^. Arachidonate 5-lipoxygenase (ALOX5) is involved in fatty acid metabolism by converting arachidonic acid into leukotrienes. BCR-ABL1 failed to induce CML in mice lacking ALOX5 due to faulty LSC self-renewal^45^. ALOX15, which encodes arachidonate 15-lipoxygenase, was found to be critical for the survival of LSCs in a murine model of BCR-ABL1-induced CML^46^. Leukotriene B4 (LTB4) is one of the products of ALOX15 and stimulates the leukotriene receptor BLT2, the expression of which is significantly increased in CML CD34^+^ stem/progenitors. Interestingly, inhibition of this pathway with the BLT2-specific inhibitor LY25528 induced apoptosis and inhibited self-renewal in CD34^+^ cells from patients with TKI-resistant blast crisis CML^47^.

### Gene regulation

The expression of genes involved in LSC survival is largely controlled at the transcriptional level through a network of nuclear factors activated downstream of signaling pathways such as the STAT3/5, FOXO3A, β-catenin, and c-MYC pathways (Fig. 2). Gene regulation is also controlled by changes in chromatin structure. Promyelocytic leukemia protein (PML), encoded by a gene involved in chromosomal translocations in leukemia, coordinates the formation of matrix-associated domains known as PML nuclear bodies, which are involved in genome maintenance through DNA repair, telomere homeostasis, and p53-associated apoptosis pathways. The role of PML in tumor suppression has been extensively investigated, and accumulating evidence indicates that PML also has roles in stem cells. Upregulation of PML induces quiescence in CML LSCs, thereby increasing their resistance to TKIs. The use of all-*trans* retinoic acid and/or arsenic trioxide could help eliminate CML LSCs by promoting exit from quiescence and active cell division^48^.

Enhancer of Zeste homolog 2 (EZH2) is a histone methyltransferase that is a component of polycomb repressive complex 2 (PRC2), which catalyzes trimethylation of histone H3 at Lys 27 (H3K27me3) to regulate gene expression. The epigenetic modification H3K27me3 is associated with the formation of heterochromatin and gene suppression. In addition to catalyzing the formation of H3K27me3, EZH2 can directly regulate gene expression. EZH2 is highly expressed in CML LSCs, and TKI treatment decreases its expression. The combination of an EZH2 inhibitor with nilotinib was found to potentiate the capacity of each single agent to eradicate CML CD34^+^ cells in a xenograft mouse model while sparing normal stem cells^49^.

Sirtuin 1 (SIRT1) is a nicotinamide adenosine dinucleotide (NAD)-dependent deacetylase involved in the removal of acetyl groups from proteins. SIRT1 is involved in a broad range of physiological functions, such as gene expression, metabolic activity, and aging^50^. Deregulation of SIRT1 in CML LSCs promotes the drug resistance and of in CML LSCs by deacetylating many transcription factors, including p53, Ku70, and FOXO1^51,52^. Pharmacological inhibition or genomic silencing of SIRT1 was found to increase cell death in CML CD34^+^ cells via an increased percentage of acetylated p53, synergizing with imatinib^53^.

### Cell survival

The BCL2 protein regulates apoptosis and was the first apoptosis modulator described to be associated with cancer. BCL2 is an important therapeutic target in hematological malignancies because it drives resistance to apoptosis and is a key feature of cancer cells that sustains tumor growth and resistance to treatment. As in many cancer types, BCL2 is highly expressed in CML, particularly in the LSC population, and is perhaps activated by PI3K/AKT and JAK/STAT activity downstream of BCR-ABL1, which supports the development of anti-BCL2 agents to deactivate resistant LSCs^54^. Inhibition of BCL2 with sabutoclax, an inhibitor of all prosurvival BCL2 family members, impaired the survival and colony-forming capacity of LSCs^55^. In addition, the potent and highly selective BCL2 inhibitor ABT-199 (venetoclax), approved for chronic lymphocytic and acute myeloid leukemias, effectively reduced the frequency of murine CML LSCs and induced apoptosis in CML patient samples in combination with TKI-mediated BCR-ABL1 inhibition, and this drug combination was also effective in leukemia stem cells from blast crisis CML patients^56^.

In a seminal proteomic study of unmanipulated CD34^+^ cells from CML patients and normal individuals by mass spectrometry, p53 and c-MYC were found to be at the center of a CML network involved in the regulation of LSCs^57^. These two well-known players in carcinogenesis, i.e., p53 and c-MYC, join forces in CML to ensure disease progression. Not surprisingly, pharmacological inhibition of p53 degradation with a small molecule reactivating p53 and inducing tumor apoptosis (RITA) combined with a BET inhibitor to block c-MYC transcription was shown to synergistically increase apoptosis in CML CD34^+^ cells with a lesser effect on normal HSCs, at least at low concentrations^57^. Another group showed that p53 activation by an MDM2 inhibitor can sensitize quiescent CD34^+^ cells to BCL2 inhibitor- and TKI-induced apoptosis^58,59^. As mentioned earlier, p53 activation through acetylation caused by SIRT1 inhibition diminished the number of CML LSCs when the SIRT1 inhibitor was used in combination with a TKI^53^. Finally, the ubiquitin ligase Fbw7 modulates c-MYC protein levels in LICs, genetic loss of c-MYC abrogates BCR-ABL1-induced disease progression, and Fbw7 regulates CML-initiating cell survival via p53 activation^60^. Collectively, these data suggest that p53 and c-MYC play critical roles in LSC maintenance and constitute important pharmacological targets for CML treatment. As discussed next, DYRK2 was identified as a single target to disarm the p53/c-MYC nodes in the CML network.

## DYRK2 regulation of leukemic stem cell self-renewal

Through study of the role of the pioneering transcription factor KLF4 in CML pathogenesis, the kinase DYRK2 was identified as a novel master regulator of a CML network involved in the self-renewal and survival of LSCs^14^. Using a mouse model based on retroviral transduction of BCR-ABL1 into bone marrow HSCs (Lin^−^Sca-1^+^c-Kit^+^CD150^+^) with conditional deletion of the *Klf4* gene and subsequent transplantation of these cells into cytoablated recipient mice, we found that loss of KLF4 led to spontaneous regression of CML-like disease associated with progressive attrition of LSCs in the bone marrow and spleen^14^. The observed ‘unstable CML disease’ was also seen when deletion of the *Klf4* gene was induced after transformation, transplantation, and establishment of myeloid leukemia cells, which suggests that regression of leukemia was not due to differential homing of *Klf4*-deficient cells into bone marrow. Although no significant alterations in cell proliferation and apoptosis were observed, analysis of purified CML LSCs (Lin^−^Sca-1^+^c-Kit^+^BCR-ABL1^+^) using two gold-standard assays to evaluate the self-renewal capacity of malignant HSCs revealed the inability of these cells to serially generate colonies in methylcellulose cultures and, most importantly, to recapitulate leukemia upon transplantation into secondary hosts, suggesting that loss of KLF4 severely abrogates self-renewal capacity. Interestingly, impairment of self-renewal similar to that in *Klf4*-deficient LSCs was reported in *Tcf1*^*–/–*^ *Lef1*^*–/–*^ CML, and one of the most strongly downregulated genes in this study was *Klf4*, suggesting that the observed effect in *Tcf1*^*–/–*^ *Lef1*^*–/–*^ CML could be mediated by loss of KLF4^61^. Because prostaglandin E1 was identified in a connectivity map analysis to elicit changes in gene expression similar to those in *Tcf1*^*–/–*^*Lef1*^*–/–*^ LSCs, and prostaglandin E1 impairs the activity of LSCs, it is possible that prostaglandins also regulate KLF4 in CML, as seen during macrophage polarization^61–63^.

**Fig. 3:**
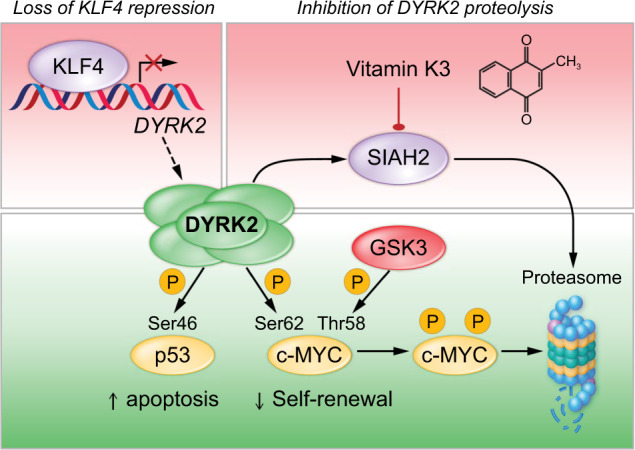
The kinase DYRK2 promotes the survival and inhibits the self-renewal of LSCs. DYRK2 has emerged as a novel target to eradicate CML LSCs. DYRK2 mediates p53 activation and c-MYC proteasomal degradation through phosphorylation. DYRK2 expression is regulated genetically by the transcriptional repressor KLF4 and by SIAH2-mediated proteasomal degradation. Pharmacological inhibition of SIAH2 with vitamin K3 (VK3) induces dose-dependent cytotoxicity associated with DYRK2 stabilization, p53 activation, and c-MYC depletion. Treatment of murine and human CML cells with VK3 has shown proof of concept that modulation of the DYRK2 protein with small molecules is a novel approach to eradicate LSCs.

Because KLF4 expression is required to sustain CML disease through the regulation of LSC self-renewal, the identification of KLF4-regulated genes in CML LSCs has the potential to uncover new targets for CML therapy. Transcriptome analysis comparing wild-type and *Klf4*-deficient LSCs yielded a list of deregulated genes involved in cell growth and proliferation, cell death and survival, and protein degradation. DYRK2 was the most strongly upregulated gene in the absence of KLF4, a result validated by immunoblotting using purified murine CML LSCs^14^. DYRK2 is a dual-specificity tyrosine-regulated kinase involved in proteasomal degradation of key cell cycle regulators (e.g., p53, c-MYC, and c-Jun)^64^. Interestingly, DYRK2 upregulation in purified CML LSCs was associated with depletion of c-MYC protein and elevated levels of p53 phosphorylated at Ser 46, and inhibition of the proteasome allowed accumulation of c-MYC protein phosphorylated at Ser 62 (the DYRK2 site) and Thr 58 (the GSK3 site). This finding suggests that DYRK2 first phosphorylates c-MYC at Ser 62, leading to phosphorylation at Thr 58 by GSK3 and subsequent proteasomal degradation (Fig. 3). Further supporting this model, tagging GFP to the endogenous *c-Myc* locus in KLF4-deficient CML LSCs showed a significant reduction in GFP-cMYC, and ectopic expression of a c-MYC mutant with an amino acid substitution at position 62 (Ala for Ser) restored the progression of CML disease in mouse models^14^. These findings are consistent with previously described functions of DYRK2 in cell cycle control through phosphorylation of c-Jun and c-MYC in HeLa cells^65^. Similar to DYRK2, although it is inhibited by BCR-ABL1, BLK inhibits self-renewal and increases apoptosis in LSCs through a pathway involving an upstream regulator, PAX5, and a downstream effector, p27^66^.

## Functions of DYRK2 in normal tissues and malignancies

**Fig. 4:**
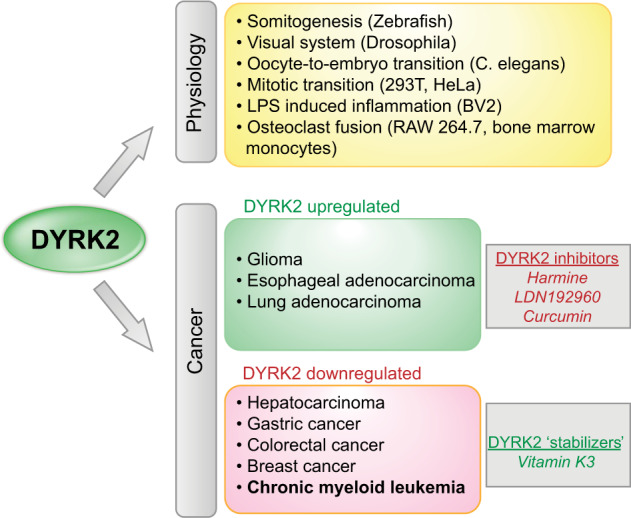
Roles of DYRK2 in normal and malignant tissues. DYRK2 is involved in physiological and malignant processes. In cancer, DYRK2 is considered to play a pro-oncogenic role at high expression levels and a potentially tumor-suppressive role with downregulation. Known small molecules that either inhibit or stabilize DYRK2 are indicated.

The DYRK family is a family of dual-specificity kinases composed of five members (DYRK1A, DYRK1B, DYRK2, DYRK3, and DYRK4) in mammals. Autophosphorylation at a tyrosine residue located in the activation loop is required for full kinase activity toward threonine and serine residues in target proteins, which are generally involved in cell survival, cell differentiation, and cell division. DYRK2 is normally localized in the cytosol, although it can translocate into the nucleus under genotoxic conditions. The physiological function of DYRK2 in cellular and tissue homeostasis is ill-defined despite its known role in cell cycle regulation (Fig. 4). DYRK2 regulates muscle differentiation during early somitogenesis in zebrafish, development of the visual system in Drosophila, and regulation of maternal-protein degradation during the oocyte-to-embryo transition in *Caenorhabditis elegans*^67–69^. In 293T and HeLa cells, DYRK2 controls the mitotic transition by mediating the phosphorylation and subsequent ubiquitination and degradation of the ATPase katanin p60 as part of an integrated complex with the EDVP E3 ligase^70^. Physiological control of mitosis may have clear implications in carcinogenesis. DYRK2 was found to inhibit the release of TNF-α and IL-1β in response to LPS in a microglial cell line through its subcellular redistribution and interactions with key signaling pathways (e.g., AKT and p38-MAPK)^71^. Finally, DYRK2 was shown to modulate osteoclast fusion through suppression of the fusion competency of follower cells by an unknown mechanism^72^. Based on this finding, it would be interesting to identify alterations in DYRK2 functions in patients suffering from altered bone homeostasis or hematopoiesis. In summary, the physiological functions of DYRK2 suggest that transient DYRK2 activation through protein stabilization may not cause damage in normal tissues.

There is evidence that DYRK2 is involved in carcinogenesis through different mechanisms, including regulation of the G1-to-S-phase transition during cell division, epithelial-mesenchymal transition, and cancer cell stemness. Overexpression of DYRK2 can be pro-oncogenic because several of its protein targets, which lead to either protein degradation or activation, are involved in cell growth and development (Table 2). DYRK2 expression in glioma correlates with the expression of E-cadherin, and its ectopic expression in glioma cell lines inhibits migration through the PI3K/AKT signaling pathway^73^. CGH analysis and STS amplification mapping revealed gene duplication and increased transcript levels of DYRK2 in esophageal and lung adenocarcinomas^74^. For malignancies in which DYRK2 expression is associated with tumor progression, the use of DYRK2 inhibitors may be an alternative therapeutic approach. Harmine, compound LDN192960, and curcumin have been described as inhibitors of DYRK2 activity that could potentially be tested for antitumor therapy (Fig. 4)^75–77^.

**Table 2:** Molecular targets of DYRK2.

Function	Phosphorylation target	Cell/tissue	Reference
Proteasomal degradation	Rpt3	Breast cancer	^84^
	MEX-5	Oocytes	^96^
	OMA-1	Oocytes	^69^
	Snail	Breast cancer, ovarian cancer	^82,97^
	hPXR	Neurons	^98^
	c-MYC	Cervical cancer, leukemia (CML)	^14,65^
	c-Jun	Cervical cancer	^14,65^
	mTOR	Breast cancer	^99^
	TBK1	Embryonic kidney	^100^
	TERT	Cervical cancer, embryonic kidney	^101^
Activation	p53	Osteosarcoma, colon cancer	^102^
	SIAH2	Osteosarcoma, embryonic kidney	^86^
	Dpysl2, Dpysl3	Neurons	^103^
	NDEL1	Neurons	^104^
Inhibition	Akt, p38-MAPK, p65 (RELA)	Neurons	^105^

Repression of DYRK2 expression has been associated with the progression of cancers such as hepatocellular carcinoma, gastric cancer, colorectal cancer, and breast cancer, and we studied this association in CML^14,78–81^. Interestingly, genomic silencing of DYRK2 increased the ability of cells from pulmonary metastases of breast cancers to form mammospheres during serial passage, an assay of self-renewal capacity, and this effect was associated with elevated KLF4 levels; however, in this case, KLF4 expression was regulated by the androgen receptor through a pathogenic DYRK2-AR-KLF4 pathway, although the mechanism by which DYRK2 activated the androgen receptor was not identified^81^. Therefore, this study and our study showed that KLF4 represses DYRK2 gene expression in CML, while in breast cancer, DYRK2 prevents androgen receptor-mediated activation of the KLF4 gene. DYRK2 can also regulate cancer invasion and metastasis by degrading Snail; therefore, shRNA silencing of DYRK2 promoted tumor invasion and metastasis in immunodeficient mice subcutaneously implanted with MCF-7 cells^82^. Further supporting a tumor-suppressive function for DYRK2 and the potential of DYRK2 stabilization as an anticancer therapy, ectopic expression of DYRK2 inhibits cell proliferation, induces cell death, and prevents tumor growth in liver cancer^83^. As part of a regulatory feedback loop, DYRK2 enhances proteasomal degradation via phosphorylation of the 19S subunit Rpt3 during the cell cycle; thus, loss of DYRK2 inhibits tumor formation by breast cancer cell lines^84^. For malignancies exhibiting downregulation of DYRK2, inhibition of proteasomal degradation and protein stabilization constitute novel therapeutic strategies.

## Pharmacological upregulation of DYRK2 abrogates self-renewal in CML LSCs

Given the deleterious effect of the DYRK2 protein on the stability of CML, a novel approach for LSC eradication based on DYRK2 protein stabilization was postulated^14^. Vitamin K3 (VK3) was tested as a proof of concept because it was shown that the ubiquitin ligase SIAH2 mediates proteasomal degradation of DYRK2 and that VK3 inhibits SIAH2 activity. As expected, VK3 induced dose-dependent cytotoxicity associated with upregulation of DYRK2, reduction of c-MYC, and induction of apoptosis in CML cell lines. VK3, also called menadione, is a synthetic compound with vitamin K activity and is chemically similar to natural vitamins K1 (phylloquinone) and K2 (menaquinone), both of which are inactive in CML cells^14^. Upregulation of DYRK2 in CML through VK3-mediated SIAH2 inhibition was further supported by biochemical assays: (a) VK3 treatment induced upregulation of prolyl hydroxylase domain 3 (PHD3), a known SIAH2 target, in addition to DYRK2; (b) c-MYC depletion was caused by VK3-mediated upregulation of DYRK2 and thus was lost by knocking out the *DYRK2* gene via CRISPR/CAS9 technology; and (c) incubation of CML cells with VK3 decreased the level of ubiquitinated DYRK2. Importantly, in vivo administration of VK3 bisulfite, a hydrophilic analog of VK3 that retains antileukemic properties, was able to reduce the frequency of LSCs, as evidenced by transplantation of whole bone marrow from leukemic mice treated with VK3 bisulfite. Most importantly for clinical translation, VK3 treatment of CD34^+^ CML cells from patients abrogated their colony-forming capacity in vitro and cleared CML blasts in a patient-derived xenograft model^14^. Notably, in vivo administration of VK3 in wild-type mice did not alter the frequency of HSCs in bone marrow, and human CD34^+^ CML cells treated with VK3 in vitro did not show significant apoptosis, suggesting that VK3 might not have toxic effects on steady-state hematopoiesis. However, the finding that vitamin K antagonists reduced the frequency of HSCs warrants further studies of VK3 in normal hematopoiesis^85^. DYRK2 and SIAH2 are mutually regulated, and it has been shown that DYRK2 phosphorylates and activates SIAH2 and that hypoxia promotes SIAH2-mediated polyubiquitination and proteasomal degradation of DYRK2^86^. Although this study was carried out using 239T cells, this finding is particularly relevant to leukemia because a hypoxic bone marrow environment may contribute to DYRK2 upregulation by SIAH2 inhibition.

## Future directions

Current data suggest that the development of LSC-specific drugs as adjuvants to TKI therapy may be a suitable approach to improve the rate of treatment-free remission in CML patients. Vitamin K3 was important for validating the role of KLF4-DYRK2 in the self-renewal of CML LSCs but might not be the appropriate drug for clinical translation because of toxicities to red blood cells, highlighting the need for novel small molecules with the same properties but enhanced safety in patients. Pharmacological stabilization of the DYRK2 protein is expected to be beneficial for CML patients, as it abrogates the self-renewal capacity of LSCs by depleting c-MYC and induces their apoptosis by activating p53^80,83,87^. The limited data on the physiological functions of DYRK2 in tissue homeostasis suggests that transient upregulation of DYRK2 may not be toxic to normal tissues, although further safety studies of identified DYRK2 stabilizers need to be conducted in preclinical studies. In addition to the on- and off-target activity of identified DYRK2 stabilizers, the effect of DYRK2 on effector immunosurveillance must be evaluated to ensure successful induction of treatment-free remission upon TKI discontinuation. In the near future, oncologists are anticipated to be able to discuss safe alternatives to reach the milestone of ‘cure’ or lack of disease recurrence after cessation of TKI therapy in CML patients in remission.
